# Impact of laparoscopic versus open surgery on humoral immunity in patients with colorectal cancer: a systematic review and meta-analysis

**DOI:** 10.1007/s00464-023-10582-0

**Published:** 2023-12-15

**Authors:** A. Bohne, E. Grundler, H. Knüttel, V. Völkel, A. Fürst

**Affiliations:** 1 Universität Regensburg, Universitätsstraße 31, 93053 Regensburg, Germany; 2 Universität Regensburg, Universitätsbibliothek Regensburg, Universitätsstraße 31, 93053 Regensburg, Germany; 3 Tumorzentrum Regensburg – Zentrum für Qualitätssicherung und Versorgungsforschung, Universität Regensburg, Am Biopark 9, 93053 Regensburg, Germany; 4 Caritas Krankenhaus St. Josef Regensburg, Klinik Für Allgemein-, Viszeral-, Thoraxchirurgie und Adipositasmedizin, Landshuter Str. 65, 93053 Regensburg, Germany

**Keywords:** Meta-analysis, Laparoscopy, Colorectal cancer, Inflammation, Surgical stress response, Cytokine

## Abstract

**Background:**

Laparoscopic surgery (LS) is hypothesized to result in milder proinflammatory reactions due to less severe operative trauma, which may contribute to the observed clinical benefits after LS. However, previous systematic reviews and meta-analyses on the impact of LS on immunocompetence are outdated, limited and heterogeneous. Therefore, the humoral response after laparoscopic and open colorectal cancer (CRC) resections was evaluated in a comprehensive systematic review and meta-analysis.

**Methods:**

Included were randomized controlled trials (RCTs) measuring parameters of humoral immunity after LS compared to open surgery (OS) in adult patients with CRC of any stage. MEDLINE, Embase, Web of Science (SCI-EXPANDED), Cochrane Library, Google Scholar, ClinicalTrials.gov and ICTRP (World Health Organization) were systematically searched. Risk of bias (RoB) was assessed using the Cochrane RoB2 tool. Weighted inverse variance meta-analysis of mean differences was performed for C-reactive protein (CRP), interleukin (IL)-6, IL-8, tumour necrosis factor (TNF)α and vascular endothelial growth factor (VEGF) using the random-effects method. Methods were prospectively registered in PROSPERO (CRD42021264324).

**Results:**

Twenty RCTs with 1131 participants were included. Narrative synthesis and meta-analysis up to 8 days after surgery was performed. Quantitative synthesis found concentrations to be significantly lower after LS at 0–2 h after surgery (IL-8), at 3–9 h (CRP, IL-6, IL-8, TNFα) and at postoperative day 1 (CRP, IL-6, IL-8, VEGF). At 3–9 h, IL-6 was notably lower in the LS group by 86.71 pg/ml (mean difference [MD] − 86.71 pg/ml [− 125.05, − 48.37], *p* < 0.00001). Combined narratively, 13 studies reported significantly lower concentrations of considered parameters in LS patients, whereas only one study reported lower inflammatory markers (for CRP and IL-6) after OS.

**Conclusion:**

The increase in postoperative concentrations of several proinflammatory parameters was significantly less pronounced after LS than after OS in this meta-analysis. Overall, the summarized evidence reinforces the view of a lower induction of inflammation due to LS.

**Supplementary Information:**

The online version contains supplementary material available at 10.1007/s00464-023-10582-0.

Colorectal cancer (CRC) is one of the leading health issues of modern times, with almost two million new cases worldwide in 2020 and a further rise expected [1]. Curative treatment still relies primarily on surgical resection, with laparoscopic and open surgery being the methods most used. While open surgery remained the “conventional” surgical approach for many decades, laparoscopic surgery has since been on the rise. Meta-analyses and systematic reviews could demonstrate better postoperative clinical outcomes after laparoscopic CRC resection, i.e., shorter hospital stay and reduced incidences of surgical wound infection and abdominal abscess [2, 3], while oncological outcomes were shown to be noninferior [4, 5] and probably even superior [6] to the open approach.

Every surgical intervention corresponds to a controlled trauma, leading to alterations in host immunity [7]. A proinflammatory reaction proportional to the extent of the surgical trauma is triggered [8] and subsequently followed by a much more pronounced compensatory anti-inflammatory reaction. Both states are associated with unwanted outcomes: hyperinflammation is related to tissue destruction, organ failure, a higher incidence of systemic inflammatory response syndrome (SIRS) [9, 10] and worse long-term survival [11], whereas the anti-inflammatory state makes the host more susceptible to infection [12]. The latter is very important in the context of cancer treatment, as suppressed immunity is known to promote metastasis formation and recurrence, hindering the (mostly) curative intent of surgical tumour resections [13].

It is hypothesised that alterations in host immunity are less pronounced in laparoscopically operated patients due to less severe surgical trauma. This milder proinflammatory reaction, followed by less pronounced immunosuppression, might in turn be an underlying mechanism contributing to the clinical benefits observed after laparoscopic surgery compared to open surgery. Systematic reviews and meta-analyses of randomized controlled trials (RCTs) evaluating immunocompetence after laparoscopic surgery do exist, but are restricted to studies published until 2012 [14, 15]. Faced with a limited and heterogenous set of studies and results of meta-analysis based on the data of just two studies, the authors themselves stated that conclusions were not possible on account of limited data availability [16].

Since the impact of surgery on host immunity in cancer remains a widely investigated topic and the status of laparoscopic surgery in colorectal cancer treatment is discussed to this day, the current article aims to provide an updated and rigorous review of the influence of laparoscopy on the humoral aspect of immunity and the proinflammatory reaction following surgical resection. The impact on cellular immunity is addressed elsewhere [17].

## Materials and methods

The Preferred Reporting Items for Systematic reviews and Meta-Analyses (PRISMA) 2020 checklist was used when writing the report [18]. The protocol of this systematic review was registered prospectively in PROSPERO under registration number CRD42021264324, with a draft search strategy published in a public repository [19].

### Eligibility criteria

Only RTCs were included in this review. The population of interest comprised patients with proven CRC of any stage. Minimally invasive (robotic and/or laparoscopic) tumour resection had to have been compared with open tumour resection in a nonemergency setting. Eligible outcomes were postoperatively measured humoral immunological parameters.

### Study identification

The following bibliographic online databases and trial registries were searched last on December 10, 2021: MEDLINE (via Ovid), Embase (via Ovid), Cochrane Database of Systematic Reviews (CDSR) and the Cochrane Central Register of Controlled Trials (CENTRAL; via Cochrane Library, Wiley), Science Citation Index Expanded (via Web of Science Core Collection), Google Scholar, ClinicalTrials.gov and the World Health Organization International Clinical Trials Registry Platform (WHO ICTRP). An initial search strategy was developed for MEDLINE and then adapted to the other databases by choosing appropriate search syntax and index terms. The search strategies aimed for high sensitivity using a broad range of synonyms and thesaurus terms. No limits such as date, language or study type were employed. The search strategy was built according to the PICO framework: population: colorectal cancer; intervention: laparoscopy; control: open surgery. Full reproducible search strategies and additional details of the searches as well as a PRISMA-S checklist [20] are contained in public repositories [21, 22]. The reference lists of relevant systematic reviews [14–16] and of the included studies were screened for further relevant studies.

Database records were imported into EndNote (version 19.3; Clarivate, London, UK). Deduplication was performed in EndNote (semiautomatic steps A–C) according to the Bramer method [23], followed by a second round employing the Systematic Review Accelerator [24]. After deduplication the records were imported into Rayyan [25] and screened for eligibility first by title and abstract, followed by a round of full-text assessment. Studies without full texts in German or English were excluded due to resource limitations. Screening was independently carried out by two authors (EG and AB), with discrepancies solved by discussion.

### Data collection process

Data were collected via the “Data Collection Form–Intervention Review–RCTs Only” of the Cochrane Collaboration [26]. The data collection sheet was adapted to the review question and included confirmation of eligibility, publication details, characteristics of the study population (age, sex, cancer stage, number of participants converted from laparoscopic to open surgery, [neo]adjuvant therapy and immunomodulatory medication), study design (allocation concealment, randomization method, number of participants included/randomized/analysed per surgical group) and surgical group characteristics (operating time, type of anaesthesia, operative methods, tumour site). In case of nonreporting of conversions, “no conversions” were assumed.

Extraction of outcome data included measuring methods, effect sizes, measures of variance and the number of participants in each group for each timepoint. Mere graphically presented data were extracted using WebPlotDigitizer version 4.6 [27]. In case of several eligible subgroups (standard or fast-track care), subgroups were pooled according to the formulas implemented in RevMan [28]. Subgroups with application of corticosteroids by default were excluded. Baseline and postoperative measurements of parameters reflecting humoral immunity up to 8 days after surgery were considered. Timeframes to group measurements were prospectively defined as 0–2 h after surgery, 3–9 h, 10–15 h, 18–30 h (equivalent to post-operative day 1 [POD1]), POD2, POD3, POD4, POD5 and POD6–8.

Data collection was carried out independently by two authors (AB and EG), with discrepancies solved by discussion. If clarifications were required, study authors were contacted once via email.

### Data analysis

All included studies were considered for narrative synthesis. Graphical display of study-level data were based on relative changes in the means per surgical group from preoperative measurements. These data were either extracted directly or calculated from absolute measurements given. If several studies contributed data within one timeframe, the minimal and maximal change from baseline was chosen per surgical approach, with the resulting corridor including all other relative changes.

To perform meta-analysis, data from at least two studies meeting the following requirements within one timeframe had to be available: data had to be either reported as mean and standard deviation (SD), or estimable (from median with interquartile and/or range) according to the method reported by Wan et al. [29]. Sensitivity analyses considering only data not estimated by this method were implemented to test the robustness of results. If reported outcome dimensions deviated from common values and those reported by other studies by at least a factor of 1000, these studies' data were not included in the meta-analysis to avoid distortion of weighting if clarification from the authors could not be obtained.

All statistical analyses were performed using Review Manager (RevMan) version 5.4.1 [28]. The mean difference (MD) accompanied by the 95% confidence interval (CI) of continuous outcomes was calculated using weighted inverse variance under assumption of the DerSimonian-Laird random-effects model. A two-sided *p* value of < 0.05 was considered significant. The presence of heterogeneity was assessed using the *Q*-statistic, *I*^2^ and *τ*^2^. Due to the low power of tests for heterogeneity, a *p* < 0.1 was defined as significant. An *I*^2^ of up to 30%, 60%, 80% or 100% was defined as indicating low, moderate, substantial, or considerable heterogeneity, respectively.

### Assessment of methodological quality and bias

Risk of bias was assessed using the Cochrane Risk of Bias (RoB)2 tool [30]. Evaluation was performed independently by two authors (AB and EG), with discrepancies solved by discussion and consulting a senior author (VV). The RoB was assessed at the study-level with the ratings “low,” “some concerns” and “high.” Intention-to-treat (ITT) analysis was not rated as appropriate to assess the effect of adhering to the intervention, hence resulting in an upgrade of the RoB. The RoB assessment also included evaluation of aspects of reporting bias (bias due to selection of the reported results and selective nonreporting), which is implemented in the RoB2 tool, and of publication bias, which is implemented in the GRADE pro GDT software [31]. The latter was used to rate the quality of the evidence of the reviews' findings for better communication of confidence in the results, with overall ratings of “high,” “moderate,” “low” or “very low quality.”

## Results

### Study selection

**Fig. 1:**
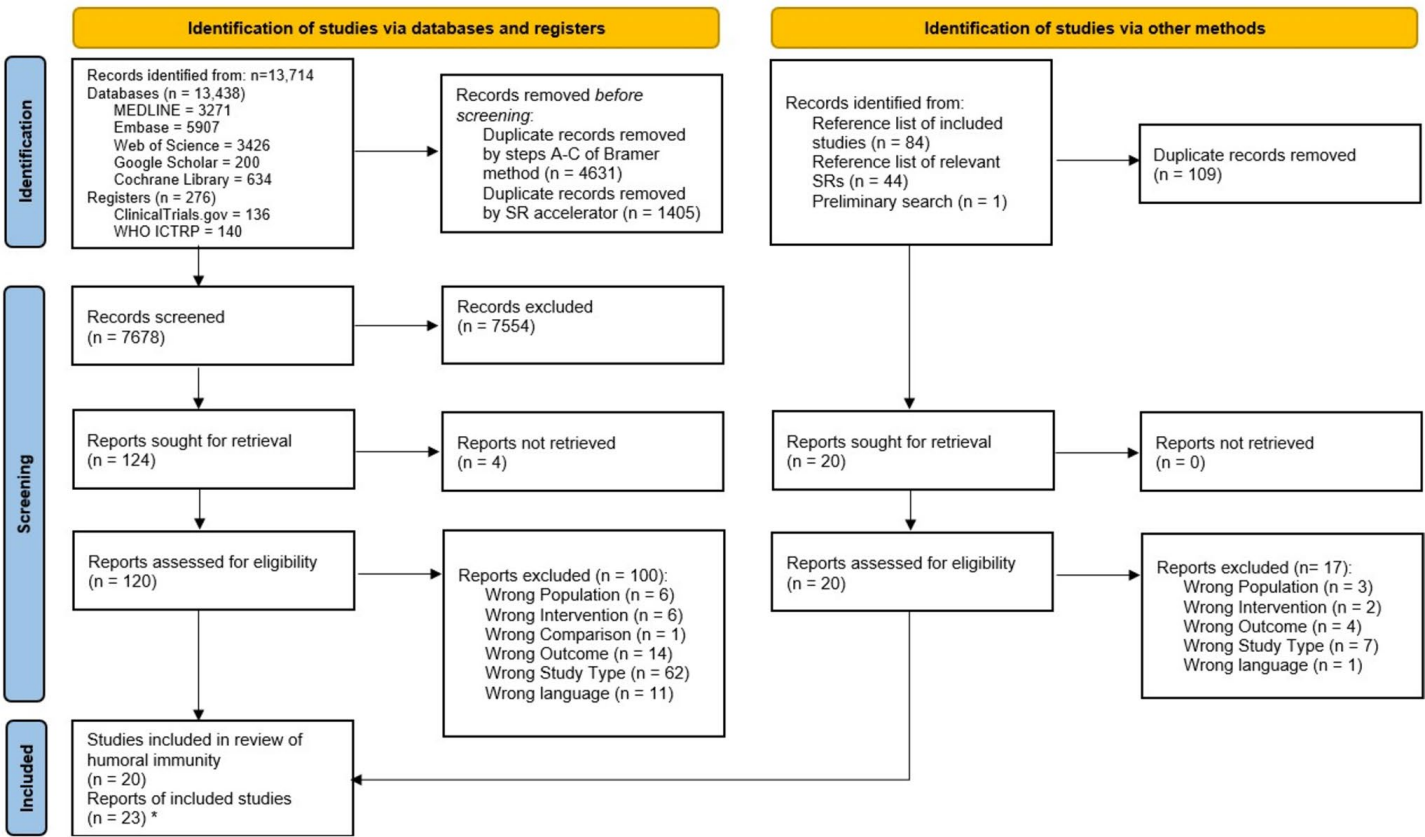
PRISMA flowchart of the study selection process; from: Page et al. [18, 372:n71]. https://doi.org/10.1136/bmj.n71. *Three times two studies were based on the same study population; therefore, 23 records were included originating from 20 studies

The systematic literature search in databases and registers yielded 13,714 records in total. Sources of records are indicated in the PRISMA flow diagram (Fig. 1) and the supplementary data. After deduplication, 7678 records were screened for eligibility according to title and abstract. Among these, 124 records were identified as potentially relevant. Full-text manuscripts were searched for, which was successful in 120 cases. Detailed justifications for exclusions of full texts are depicted in Fig. 1, with 20 reports remaining for inclusion. The citation search of these included studies yielded two further eligible reports. Citation searching in recent relevant meta-analyses and systematic reviews [14–16] identified by an initial scoping review following the work of Völkel et al. [32] produced no new records. The study of Zhao et al. [33] could not be included due to its full-text language. One report was directly and only identified by the initial scoping review. Because of identical study populations (judged by population characteristics, authors, year of publication), overall, 23 reports correspond to 20 studies included in this review of humoral immunity.

### Study characteristics

The characteristics of included studies as well as the RoB assessment can be found in Table 1; more detailed characteristics of included studies are published in a public repository [22]. Overall, data from 1131 participants were included in this review of humoral parameters. Most commonly, results from patients with Union for International Cancer Control (UICC) tumour stage II were reported, with 182 and 171 participants in the laparoscopic (LS) and open surgery (OS) groups, respectively. No study reported significant differences regarding population characteristics or significantly differing baseline immunological parameters between LS and OS. Conversions to the open approach occurred in 13 trials, four trials reported no conversions, and the remaining three trials did not give information. Nine studies reported resections of colon and rectal cancer, eight studies performed colonic resections only and three studies carried out solely rectal resections.

**Table 1:** Characteristics of included studies including numbers of participants giving information as to inclusion (yes) or exclusion (no) of UICC stage IV colorectal cancer, the localizations of resections performed, overall risk of bias rating, and the outcomes assessed by study authors which are being discussed in this publication

First author	Year of publication	*n*	LS	OS	Inclusion of stage IV	Colon and/or rectal resections	Risk of bias	Outcomes evaluated
Delgado [34]	2001	97	39	58	Yes	Colorectal	Some concerns	IL6, CRP
Duque [35]	2019	37	18	19	n.a	Colorectal	Some concerns	IL6, IL8, TNFα, CRP, VEGF
Hasegawa [36]	2003	50	24	26	No	Colorectal	Some concerns	IL6, CRP
Hewitt [37]	1998	16	8	8	No	Colorectal	Some concerns	IL6
Kim [38]	2011	57	38	19	No	Colon	Low	IL6, CRP, VEGF
Kvarnström [39, 40]	2012/2013	24	12	12	n.a	Rectal	High	IL6, IL8, TNFα, CRP
Laforgia [41]	2016	14	7	7	Yes	Colorectal	Some concerns	IL6, CRP
Leung [42]	2000	34	17	17	No	Rectal	Some concerns	IL6, TNFα, CRP
Ordemann [43]	2001	40	20	20	No	Colorectal	Some concerns	IL6, TNFα
Pascual [44]	2010	120	60	60	No	Colorectal	High	IL6, VEGF
Schwenk [45]	2000	60	30	30	Yes	Colorectal	Some concerns	IL6, CRP
Stage [46]	1997	29	15	14	Yes	Colon	Low	IL6, CRP
Straatman [47]	2018	79	42	37	n.a	Colorectal	Some concerns	CRP
Tsimogiannis [48, 49]	2011/2012	40	20	20	No	Colon	Low	IL6, TNFα, CRP
Veenhof [50]	2011	40	22	18	Yes	Rectal	Some concerns	IL6, IL8, CRP
Veenhof [51]	2012	79	42	37	n.a	Colon	Some concerns	IL6, CRP
Vignali [52]	2009	26	13	13	Yes	Colon	Some concerns	IL6, IL8, TNFα, CRP
Wang [53]	2012	163	80	83	No	Colon	Low	IL6, CRP
Wu [54, 55]	2003/2004	26	12	14	No	Colon	Low	IL6, IL8, TNFα, CRP, VEGF
Zhu [56]	2017	100	50	50	No	Colon	Some concerns	TNFα, CRP

*n.a.* no information available, *n* total study population, *LS* study population in laparoscopic group, *OS* study population in open group

The risk of bias was rated as low in five studies. In 13 studies the RoB was assessed to be of some concern and two studies were labelled with a high risk of bias. Unknown allocation concealment was the main reason to uprate the RoB arising from the randomization process. A common reason to uprate RoB due to deviations from the intended interventions were ITT analysis methods in case of conversions, affecting seven studies. The individual judgements per domain can be taken from Table S4 published at the publicly accessible repository [22].

### Results of meta-analysis and systematic review

Results of the narrative syntheses of all studies can be found in the public repository as Table S2 [22]. For graphical display of postoperative development, 17 studies were eligible. Ordemann et al. [43] and Schwenk et al. [45] only reported medians with 95% CI, Stage et al. [46] only presented medians without measures of variance, thus not allowing for estimation of means, and were hence excluded from graphical display.

Thirteen studies were included in the meta-analyses. Among these, four studies reported median with range and/or interquartile range; hence mean and SD were estimated. Seven studies could not be considered, either due to missing units [53], a data format not allowing transformation to mean and SD [43, 45, 46], missing estimates of variance [52] or missing reporting of absolute values [50, 51]. Hence, pooling of data were possible for CRP, IL-6, IL-8, VEGF and TNFα.

### C-reactive protein

Seventeen studies measured CRP concentrations in peripheral blood, with nine studies reporting significantly lower concentrations after LS up to POD5. The study by Stage et al. [46] was the only one to state lower CRP values after OS for POD1 and POD3.

**Fig. 2:**
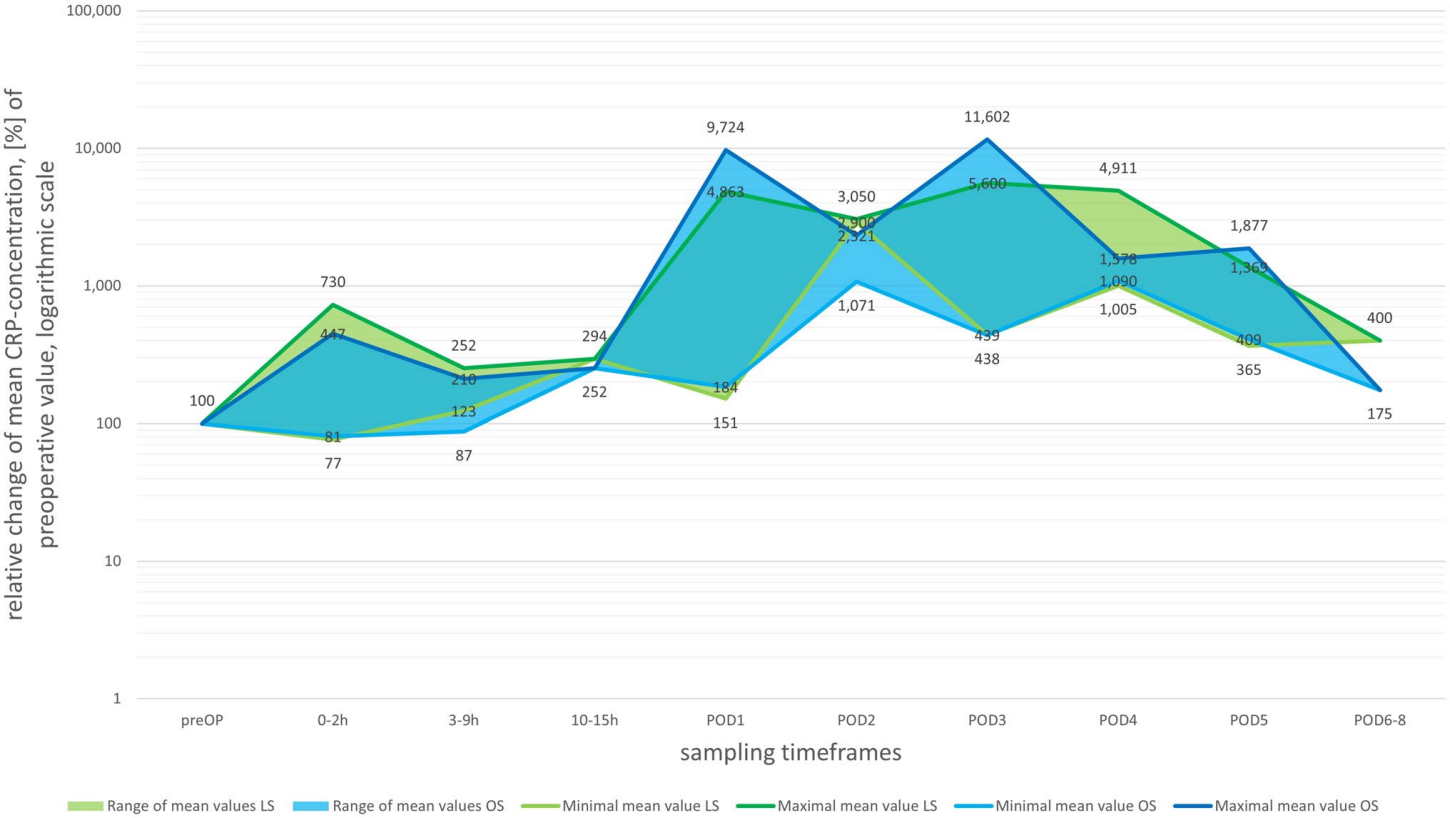
Postoperative progression of relative mean CRP concentration based on preoperative values set at 100%, numbers given are minima and maxima per timepoint stratified for surgical group

An overall increase in postoperative CRP relative to preoperative values was reported by all studies (Fig. 2). Peak values were reached at POD1 and POD3 by both the OS (97.24- and 116.02-fold increase) and the LS group (48.63- and 56.00-fold increase), although all studies reported a more pronounced rise after OS. A subsequent drop in relative CRP concentrations can be observed from POD4 onwards. During the whole observation period of up to 8 days after surgery, CRP values remained above the preoperative measurements.

**Fig. 3:**
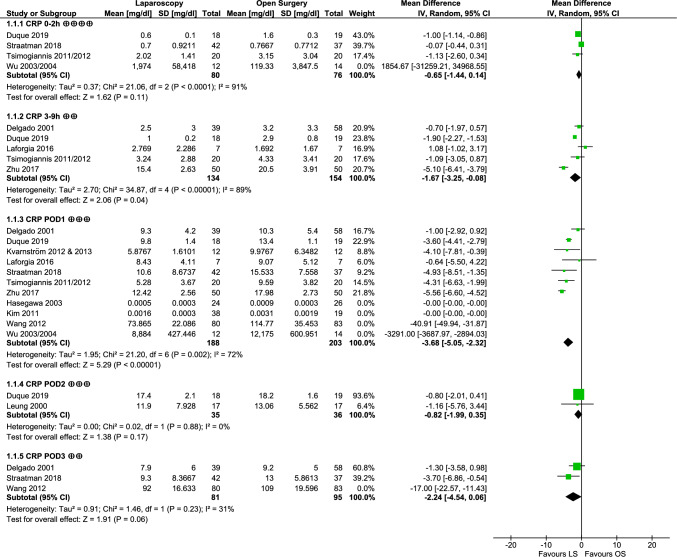
Forest plot depicting meta-analysis of CRP; GRADE quality of evidence rating is indicated by ⊕ (very low), ⊕⊕ (low), ⊕⊕⊕ (moderate), ⊕⊕⊕⊕ (high); numerical data of Hasegawa et al. [36], Kim et al. [38] and Wu et al. [54, 55] reported CRP values deviating by factor 1000 from values reported by other studies and were therefore excluded from meta-analysis; numerical data of Wang et al. [53] were not included in meta-analysis due to missing reporting of units (numbers depicted in respective data tables)

Meta-analysis results of CRP are shown in Fig. 3. There was no significant difference between the two surgical approaches observed during the first 0–2 h after surgery (MD − 0.65 mg/dl [− 1.44, 0.14], *p* = 0.11). However, 3–9 h after surgery, CRP concentrations were shown to be significantly lower after laparoscopy (MD − 1.67 mg/dl [− 3.25, − 0.08], *p* = 0.04). Likewise, CRP was significantly lower at POD1 after LS (MD − 3.68 mg/dl [− 5.05, − 2.32], *p* < 0.00001). Such significant differences could not be observed for the subsequent days (POD2: MD − 0.82 mg/dl [− 1.99, 0.35], *p* = 0.17; POD3: MD − 2.24 mg/dl [− 4.54, 0.06], *p* = 0.06). Heterogeneity was substantial at POD1 (*I*^2^ = 72%, *p* = 0.002) and considerable for 0–2 h (*I*^2^ = 91%, *p* < 0.0001) and 3–9 h (*I*^2^ = 89%, *p* < 0.00001).

Summarizing these findings, CRP values are probably slightly decreased after LS. Lower CRP concentrations and milder increases are outcomes considered favourable.

### Interleukin 6

Narrative synthesis of 18 studies showed significantly lower concentrations of IL-6 after LS in the early postoperative period up to POD1 reported by 11 studies. After this timeframe, only Wang et al. [53] reported significantly lower IL-6 in patients after laparoscopic resection. Again, only Stage et al. [46] reported higher concentrations in the LS group at POD1.

**Fig. 4:**
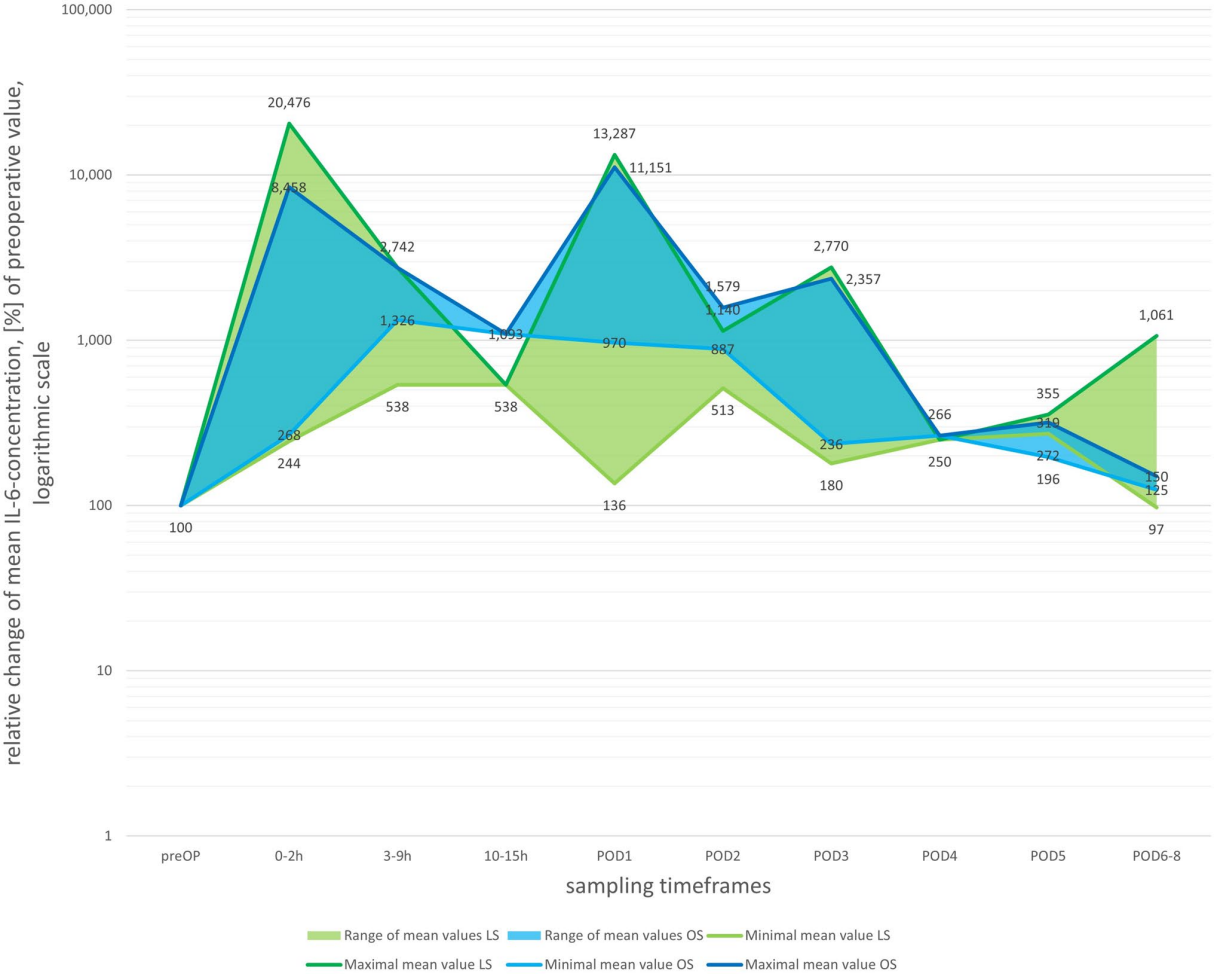
Postoperative relative progression of IL-6 based on preoperative values

Generally, studies reported rising IL-6 after both LS and OS (Fig. 4). A peak in values relative to the preoperative measurements can be observed immediately during the first 2 h after surgery (204.76-fold after LS, 84.58-fold after OS) and at POD1 (132.87-fold after LS, 111.51-fold after OS). The laparoscopic group of Laforgia et al. [41] reached concentrations comparable to preoperative values (LS POD6–8: 97%).

**Fig. 5:**
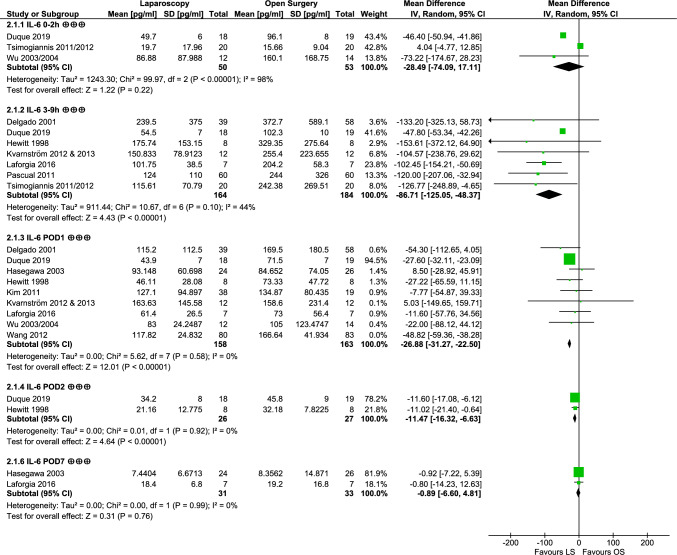
Forest plot depicting meta-analysis of IL-6; GRADE quality of evidence rating is indicated by ⊕ (very low), ⊕⊕ (low), ⊕⊕⊕ (moderate), ⊕⊕⊕⊕ (high); numerical data of Wang et al. [53] were not included in meta-analysis due to missing reporting of units (numbers depicted in respective data tables)

In meta-analysis (Fig. 5), no significant difference was observed for the first timeframe (0–2 h: MD − 28.49 pg/ml [− 74.09, 17.11], *p* = 0.22), but after 3–9 h, mean concentrations of IL-6 were significantly lower after LS by 86.71 pg/ml (MD − 86.71 pg/ml [− 125.05, − 48.37], *p* < 0.00001). Also, at POD1 (MD − 26.88 pg/ml [− 31.27, − 22.50], *p* < 0.00001) and POD2 (MD − 11.47 pg/ml [− 16.32, − 6.63], *p* < 0.00001), IL-6 was lower after LS. This difference was not present at POD6-8 according to the available data (− 0.89 pg/ml [− 6.60, 4,81], *p* = 0.76). Heterogeneity was mostly low, but considerable at 0–2 h (*I*^2^ = 98%, *p* < 0.00001) and moderate at 3–9 h (*I*^2^ = 44%, *p* = 0.10).

Overall, IL-6 values are probably lower in patients after laparoscopy compared to OS. As IL-6 is a proinflammatory cytokine, lower concentrations are favoured.

### Interleukin 8

Narrative review of the results of five studies indicated significantly lower IL-8 concentrations for 0–2 h and 3–9 h in the LS group by Duque et al. [35] and Wu et al. [54, 55], but significance was not reached at later timeframes. No study found higher concentrations after LS.

The progression chart (Fig. S1, supplement) of IL-8 shows an initial postoperative rise of relative IL-8 concentrations, considerably more prominent after OS (58.95-fold; LS: twofold at 0–2 h), whereas Kvarnström et al. [39, 40] reported a drop in IL-8 values after LS in the early postoperative period (16% at 0–2 h, 50% at POD1). Return to preoperative dimensions could be observed for all surgical groups in the later postoperative period until POD6–8.

Meta-analysis (Fig. S2, supplement) demonstrated significantly lower concentrations of IL-8 after laparoscopy, with a concentration pronouncedly lower by 72.59 pg/ml after LS compared to OS at 0–2 h (0–2 h: MD − 72.59 pg/ml [− 77.53, − 67.65], *p* < 0.00001; 3–9 h: MD − 21.24 pg/ml [− 24.10, − 18.38], *p* < 0.00001; POD1: − 4.32 pg/ml [− 6.29, − 2.35], *p* < 0.0001). Heterogeneity was low throughout.

Overall, IL-8 concentrations may be lower after LS compared to OS, with lower concentrations representing a superior outcome due to the proinflammatory domain of this interleukin.

### Tumour necrosis factor alpha

Synthesised narratively, Ordemann et al. [43] reported significantly lower concentrations after LS for 0–2 h and 3–9 h. This was again reported by Ordemann et al. [43] and Duque et al. [35] at POD2. No study found significantly lower concentrations after OS. Wu et al. [54, 55] consistently found results below their detectable limit of 25 pg/ml, not allowing statements regarding differences in concentrations.

The postoperative changes in TNFα (Fig. S3) in the included studies were less prominent and clear: minimal and maximal levels were both reported 3–9 h after surgery by different studies (LS: 0.8-fold decrease to 1.63-fold increase; OS: 0.82-fold decrease to 1.78-fold increase). Convergence to preoperative values was observed during the later postoperative period.

Meta-analysis (Fig. S4) at 0–2 h after surgery yielded TNFα levels not significantly differing between the groups (MD − 1.25 pg/ml [− 4.24, 1.74], *p* = 0.41), nor did they differ at POD1 (MD − 3.9 pg/ml [− 9.02, 1.22], *p* = 0.14). However, 3–9 h after surgery, the mean concentration of TNFα was significantly lower after LS by 7.25 pg/ml (MD − 7.25 pg/ml [− 13.04, − 1.47] *p* = 0.01). Heterogeneity was substantial (0–2 h: *I*^2^ = 77%, *p* = 0.04) to considerable for all analyses (3–9 h: *I*^2^ = 95%, *p* < 0.00001, POD1: *I*^2^ = 90%, *p* < 0.00001).

Overall, TNFα concentrations are probably lower in patients after LS, which is a favourable outcome due to this parameter's proinflammatory nature.

### Vascular endothelial growth factor

Summarizing the findings of the studies, Duque et al. [35] and Pascual et al. [44] found significant results for 3–9 h, POD2 and POD4, with lower concentrations of VEGF after LS. No study reported higher VEGF after LS compared to OS.

On the whole, VEGF concentrations were reported to rise postoperatively, with most pronounced serum levels at POD1 (1.79-fold after LS, 2.33-fold after OS) and POD4 (181-fold after LS and 2.29-fold after OS), although Kvarnström et al. [39, 40] stated decreasing values at 0–2 h after surgery (LS 65%, OS 95%). The VEGF concentration remained elevated in both groups during the available observation period (Fig. S5).

Meta-analysis of VEGF (Fig. S6) at 0–2 h did not result in a significant difference between the groups (MD − 186.82 pg/ml [− 418.20, 44.56], *p* = 0.11), whereas a significantly lower concentration of 303.15 pg/ml in the LS group compared to the OS group was present at POD1 (MD − 303.15 pg/ml [− 431.62, − 174.67], *p* < 0.00001). Substantial heterogeneity was present at 0–2 h (*I*^2^ = 66%, *p* = 0.09).

To summarize, concentrations of systemic VEGF are probably lower after LS. Identical to other proinflammatory cytokines, a lower VEGF concentration is favoured due to it indicating a lesser inflammatory reaction.

### Reporting biases

A risk of bias due to selective nonreporting was found for CRP measurements by Kvarnström et al. [39, 40]. Although stated in the methods section for all parameters, results for two timepoints were not given. One study was seen to be at risk of bias concerning selection of the reported results: Pascual et al. [44] reported differing timepoints of measurements in the protocol and the manuscript.

### Certainty of evidence

Confidence in the estimates of effect for TNFα were moderate, being limited by imprecision due to small sample sizes or due to inconsistency because of relevant heterogeneity. The CRP effect estimates were rated to be of low to high-quality. Reasons to downrate were high risks of selection bias due to possible repeated measurements, high heterogeneity and/or limited study populations with insufficient statistical power. The quality of evidence for IL-6 was rated to be moderate for all estimates of effect. Reasons to downrate were inconsistency due to high heterogeneity, high RoB due to selective reporting or a limited overall study population contributing to the analysis. Concerning IL-8, the rating was found to be moderate or low due to limited study populations in all cases and because of a high RoB in the context of non-protocol interventions. Results for analyses of VEGF were evaluated to be of moderate quality due to imprecision.

## Discussion

Generally, rising concentrations of proinflammatory mediators were observed after laparoscopic as well as open surgery, which was most pronounced in the early postoperative period in both surgical groups. This is in concordance with previous findings from other studies [57–59].

The current meta-analysis found significant differences, with lower concentrations of proinflammatory parameters after laparoscopy. These differences were present in all parameters evaluated and seen consistently during the early postoperative period up to POD1, possibly indicating that different attenuation of immunity is most prominent in the immediate postoperative period. When narratively summarizing all given evidence, a less pronounced proinflammatory reaction is still seen: after LS, significantly lower markers of inflammation were reported by the majority of studies (13 studies finding significant differences favouring LS and six not reporting differences between surgical approaches), whereas only Stage et al. [46] stated results favouring the open approach.

Clinical trials have repeatedly shown superior short-term outcomes after laparoscopy, with fewer anastomotic leaks and infections, leading to shorter hospital stay, faster recovery and reduced perioperative morbidity and mortality [60, 61]. Elevated CRP and IL-6 indicate postsurgical infection [62, 63], while CRP concentration correlates with the incidence of anastomotic leakage [64]. In the present analysis, both parameters were significantly reduced in patients receiving laparoscopy, therefore supporting the hypothesis that a milder activation of proinflammatory processes may be the reason for the beneficial short-term outcomes seen after LS.

Besides these immediate benefits, laparoscopy has not just repeatedly been proven to be noninferior regarding oncological long-term outcomes, but recent trials even indicate superiority of LS over OS in terms of long-term survival and metastasis formation [65–68]. Although this review was limited to a timeframe of up to 8 days after surgery, the most vulnerable phase determining long-term oncological outcomes is indeed directly during and after surgery [13]. Surgical trauma triggers healing responses promoting tumour cell migration, spread and angiogenesis. Moreover, manipulation of cancerous tissue is known to cause tumour cell seeding [13, 69]. Therefore, it is very important to limit excessive postoperative concentrations of inflammatory mediators: TNFα, IL-8 and VEGF are key contributors to tumorigenesis, promoting a proinflammatory state aiding tumour growth as well as cell migration and neoangiogenesis [69–71]. Interleukins generally play a crucial role in CRC [72]: IL-6 facilitates angiogenesis, migration and proliferation [73, 74]; high serum IL-8 contributes to growth and progression of CRC [75] and is even associated with resistance to chemotherapy, an important pillar of CRC treatment besides surgery [76–78]. The current results indicate lower systemic concentrations of these parameters after LS, providing a possible explanation for the reported beneficial oncological outcomes.

### Strengths and limitations

This review included data of 1131 participants from 20 RCTs on the humoral immunological impact of CRC surgery, thus representing the most comprehensive data synthesis to date. Particularly because only RCTs were included, the confidence in the estimates of effect could mostly be rated as moderate or even high. Another strength of this review lies in the rigorous search strategy applied, which reduces the risk of post-publication bias and enables a thorough overview of the topic. In contrast to previous meta-analyses, more differentiated timeframes were used for outcome grouping, while still reaching an optimal information size for several analyses.

It is important to note that mean values of CRP (3–9 h, POD1), IL-8 (POD1) and TNFα (3–9 h) showed significant differences, but these differences were not very pronounced. Although there is no common cut-off value to indicate the smallest effect size of interest and lower concentrations are generally favourable, the clinical relevance of such observed differences is unclear.

This review faces limitations resulting from included studies. Several studies had only small sample sizes. Humoral parameters as well as the sampling timepoints chosen showed high heterogeneity, rendering synthesis of results a challenging task. This resulted in some meta-analyses not reaching the optimal information size or an inability to perform meta-analysis for parameters originally planned. Study quality was satisfactory overall, although two studies [40, 44] show hints of reporting bias, which might impact on this reviews' results for CRP and IL-6. Due to the scarcity of prospective protocols corresponding to included studies, assessment of risk of bias due to selection of the reported results was limited and judgements were made based on the consistency of reporting between the methods and results sections [79].

Sensitivity analyses restricted to nonestimated data mostly yielded robust results, without changes in the level or direction of effects. However, for CRP at 0–2 h, the level of significance changed from nonsignificance to significantly lower CRP after LS (MD − 1.0 mg/dl [− 1.14, − 0.86], *p* < 0.00001). Testing for the overall effect for IL-8 at POD1 under conditions of this sensitivity analysis changed the result, leading to nonsignificance (MD − 5.73 pg/ml [− 21.3; 9.84], *p* = 0.47). Other results were not influenced.

Further high-quality studies with higher power focusing on clinically established parameters and sampling timepoints as well as provision of prospective study protocols would be desirable for future research. Also, as none of the included studies assessed the impact of robotic techniques on postoperative immunocompetence, it is the task of future research to shed further light on this topic of increasing interest.

## Concluding remarks

Altogether, a less pronounced proinflammatory reaction mediated by soluble effector molecules was seen after laparoscopic surgery compared to open surgery in this meta-analysis and systematic review. Therefore, summarized evidence of this review supports the view of a lower induction of inflammation by laparoscopic surgery, probably providing an explanatory model for the observed clinically superior short- and long-term outcomes after laparoscopic surgery.

### Supplementary Information

Below is the link to the electronic supplementary material.

Supplementary file1 (JPG 337 kb)

Supplementary file2 (EPS 828 kb)

Supplementary file3 (JPG 402 kb)

Supplementary file4 (EPS 930 kb)

Supplementary file5 (JPG 415 kb)

Supplementary file6 (EPS 662 kb)

Supplementary file7 (DOCX 31 kb)

Supplementary file8 (PDF 174 kb)

Supplementary file9 (DOCX 45 kb)

## Data Availability

Data supporting this article are available from a public repository ([21, 22]).
