# Supplementary figures and images for: Impact of laparoscopic versus open surgery on humoral immunity in patients with colorectal cancer: a systematic review and meta-analysis
Source: Surg Endosc. 2023 Dec 15;38(2):540–53. doi: 10.1007/s00464-023-10582-0 (PMC10830603; doi:10.1007/s00464-023-10582-0)

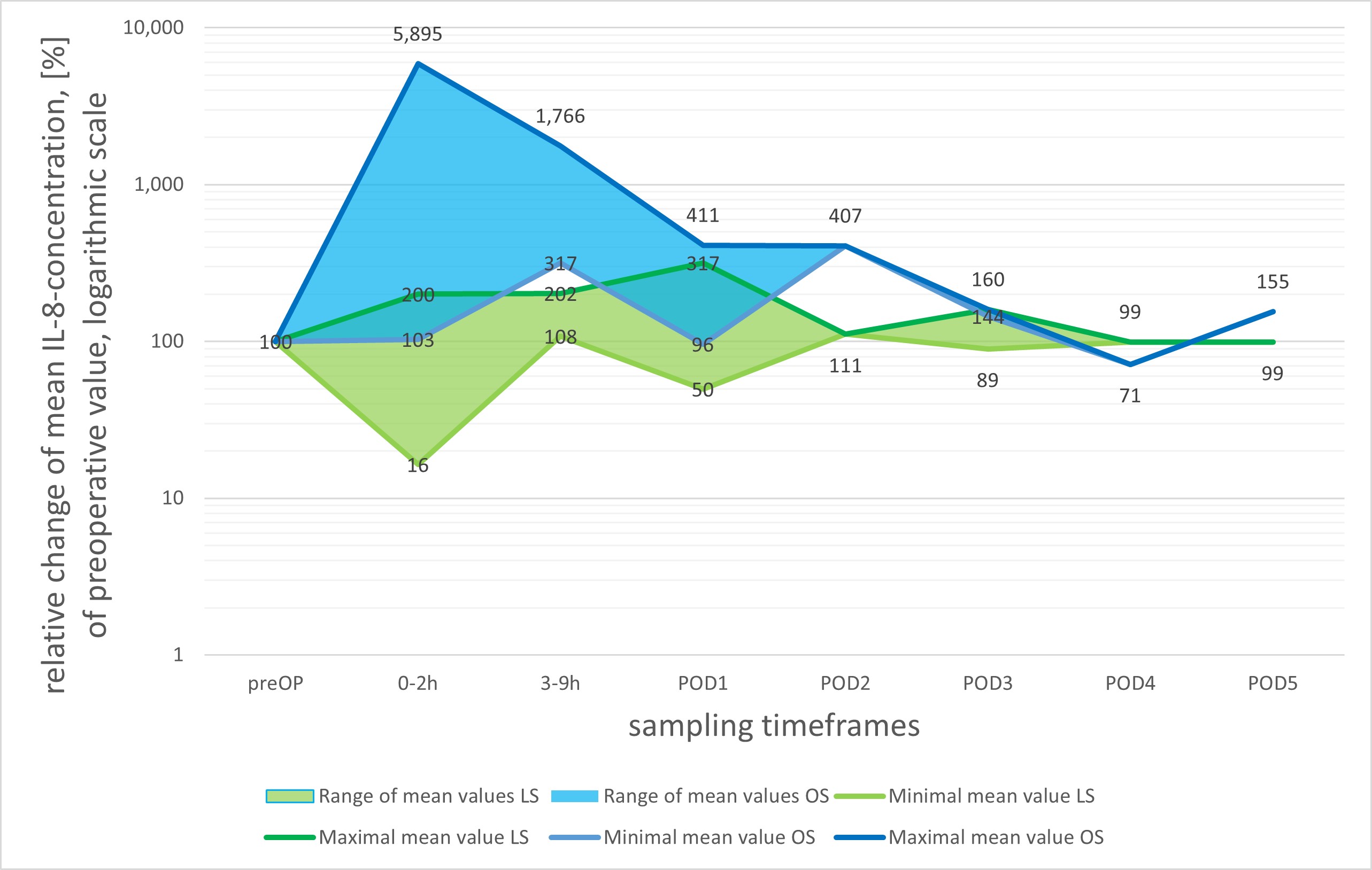

Supplement: Supplementary file 1 — Supplementary file1 (JPG 337 kb) [file 464_2023_10582_MOESM1_ESM.jpg]

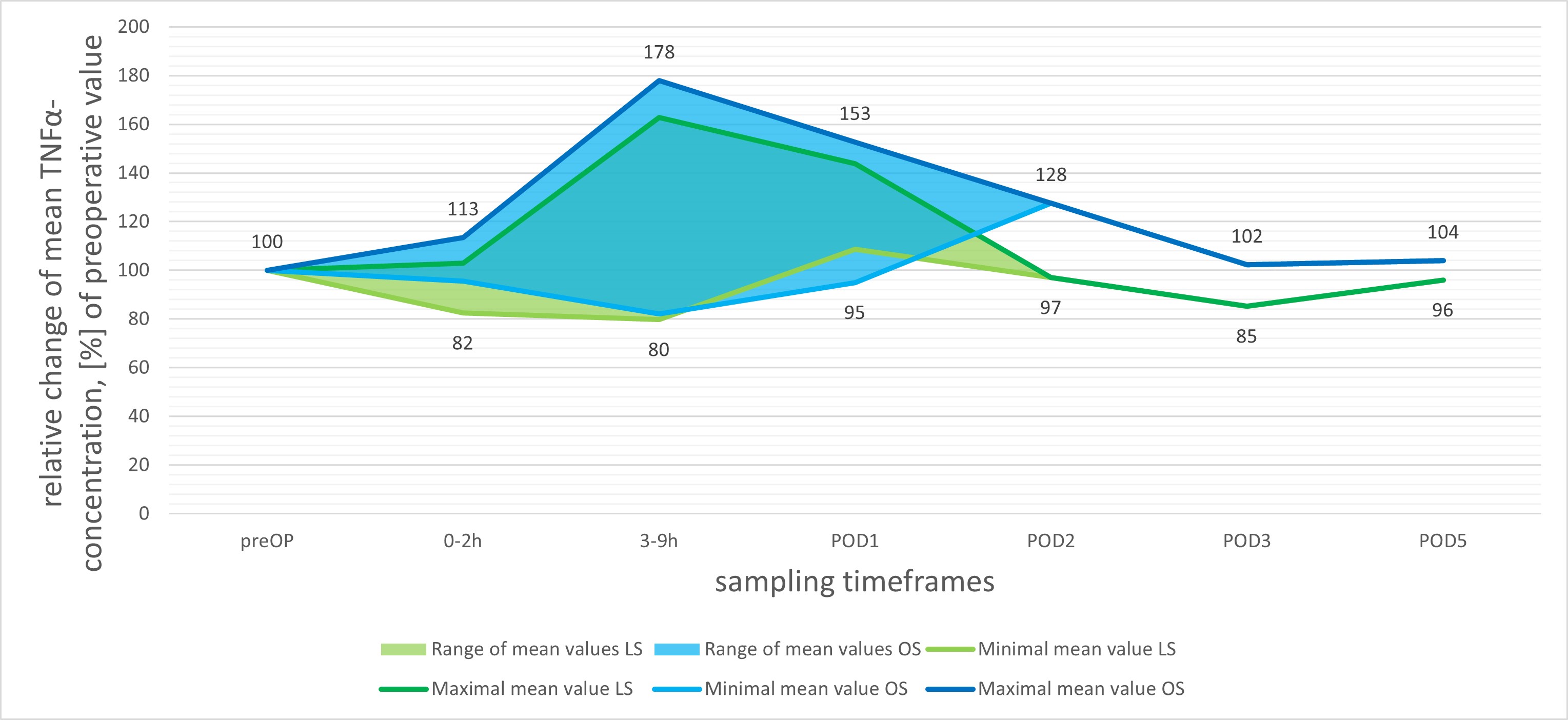

Supplement: Supplementary file 3 — Supplementary file3 (JPG 402 kb) [file 464_2023_10582_MOESM3_ESM.jpg]

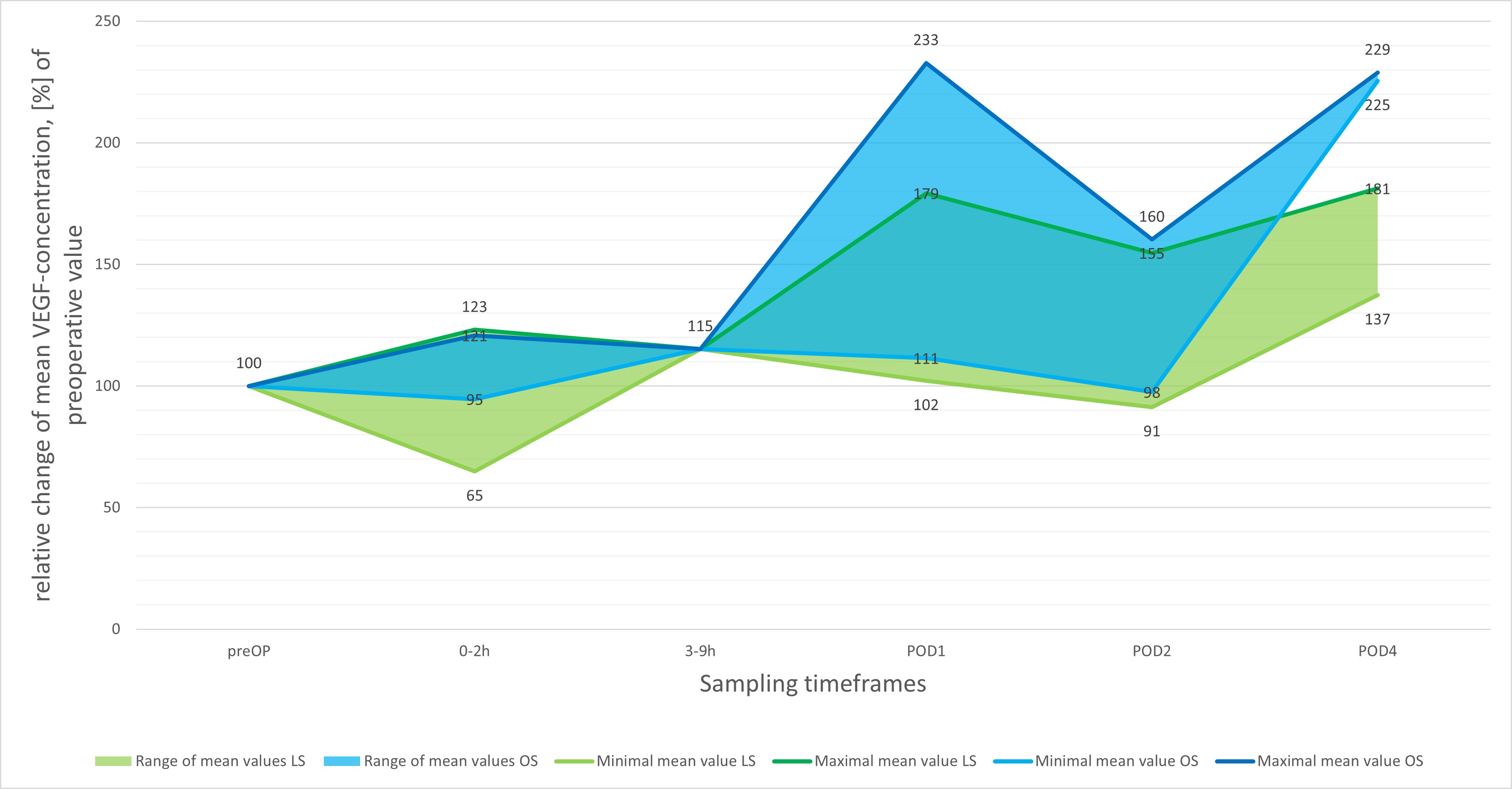

Supplement: Supplementary file 5 — Supplementary file5 (JPG 415 kb) [file 464_2023_10582_MOESM5_ESM.jpg]
