# Supplementary material for: Impact of laparoscopic versus open surgery on humoral immunity in patients with colorectal cancer: a systematic review and meta-analysis
Source: Surg Endosc. 2023 Dec 15;38(2):540–53. doi: 10.1007/s00464-023-10582-0 (PMC10830603; doi:10.1007/s00464-023-10582-0)
Supplement: Supplementary file 9 — Supplementary file9 (DOCX 45 kb) [file 464_2023_10582_MOESM9_ESM.docx]

Table S1: Characteristics of included studies; Ex = exclusion of tumour stage, n.a. = no information available, LFT = laparoscopy with fast-track care, OFT = open surgery with fast-track care; eligibility for the following analyses: MA = meta-analysis, NA = narrative analysis, G = graphical display; ^a^ mean und range; ^b^ median and range; ^c^ median and 25th-75th IQR; ^d^ median and 95% confidence interval

*data of the following studies or parameters could not be used for analyses: Hasegawa, Kim and Wu all reported unrealistic dimensions for CRP and were excluded from CRP meta-analysis, Wang reported no units and therefore being excluded from meta-analysis of IL6 and CRP

Table S2: Table of narrative results, referenced studies being: 1 Delgado2001, 2 Duque2019, 3 Hasegawa2003, 4 Hewitt1998, 5 Kim2011, 6 Kvarnström2012/13, 7 Laforgia2016, 8 Leung2000, 9 Ordemann2001, 10 Pascual2011, 11 Schwenk2000, 12 Stage1997, 13 Straatman2018, 14 Tsimogiannis2011/12, 15 Veenhof2011, 16 Veenhof2012, 17 Vignali2009, 18 Wang2012, 19 Wu2003/04, 20 Zhu2017

|  | | | |  |  |  |
| --- | --- | --- | --- | --- | --- | --- |
|  |  |  |  |  |  |  |
|  | 0-2h | | 3-9h | | POD1 | |
|  | Number of contributing studies (reference) | Result (MD [95% CI]) | Number of contributing studies (reference) | Result (MD [95% CI]) | Number of contributing studies (reference) | Result (MD [95% CI]) |
| CRP | 2 (2, 14) | -1.00 [-1.14, -0.86] |  |  | 5 (1, 2, 7, 14, 20) | -3.46 [-5.09, -1.82] |
| IL-6 |  |  | 6 (1, 2, 4, 7, 10, 14) | -87.19 [-128.99, -45.39] | 6 (1, 2, 3, 4, 7, 19) | -27.08 [-31.48, -22.67] |
| IL-8 |  |  |  |  | 2 (2, 19) | -5.73 [-21.30, 9.84] |
| TNFa |  |  | 3 (2, 14, 20) | -8.72 [-15.48, -1.96] | 3 (2, 14, 20) | -5.59 [-11.80, 0.63] |
| VEGF |  |  |  |  | 2 (2, 19) | -311.57 [-441.30, -181.84] |

Table S3: Sensitivity analyses of meta-analyses; referenced studies being: 1 Delgado2001, 2 Duque2019, 3 Hasegawa2003, 4 Hewitt1998, 5 Kim2011, 6 Kvarnström2012/13, 7 Laforgia2016, 8 Leung2000, 9 Ordemann2001, 10 Pascual2011, 11 Schwenk2000, 12 Stage1997, 13 Straatman2018, 14 Tsimogiannis2011/12, 15 Veenhof2011, 16 Veenhof2012, 17 Vignali2009, 18 Wang2012, 19 Wu2003/04, 20 Zhu2017

|  | | | | | | | |
| --- | --- | --- | --- | --- | --- | --- | --- |
|  |  | Randomization | Intervention deviattion | Missing outcome data | Measurement bias | Selective reporting | Overall RoB |
| Delgado | 2001 |  |  |  |  |  |  |
| Duque | 2019 |  |  |  |  |  |  |
| Hasegawa | 2003 |  |  |  |  |  |  |
| Hewitt | 1998 |  |  |  |  |  |  |
| Kim | 2011 |  |  |  |  |  |  |
| Kvarnström | 2012&2013 |  |  |  |  |  |  |
| Laforgia | 2016 |  |  |  |  |  |  |
| Leung | 2000 |  |  |  |  |  |  |
| Ordemann | 2001 |  |  |  |  |  |  |
| Pascual | 2010 |  |  |  |  |  |  |
| Schwenk | 2000 |  |  |  |  |  |  |
| Stage | 1997 |  |  |  |  |  |  |
| Straatman | 2018 |  |  |  |  |  |  |
| Tsimogiannis | 2011 & 12 |  |  |  |  |  |  |
| Veenhof | 2011 |  |  |  |  |  |  |
| Veenhof | 2012 |  |  |  |  |  |  |
| Vignali | 2009 |  |  |  |  |  |  |
| Wang | 2012 |  |  |  |  |  |  |
| Wu | 2003 & 04 |  |  |  |  |  |  |
| Zhu | 2017 |  |  |  |  |  |  |

Table S4: Risk of Bias table, showing ratings for each domain; green = low risk, yellow = some concerns, red = high risk
